# The impact of COX-2 on invasion of osteosarcoma cell and its mechanism of regulation

**DOI:** 10.1186/1475-2867-14-27

**Published:** 2014-03-25

**Authors:** Xing Wu, Ming Cai, Fang Ji, Lie-ming Lou

**Affiliations:** 1 Department of Orthopaedics, Shanghai tenth People’s Hospital, Tongji University School of Medicine, No.301 Middle Yanchang Road, Shanghai 200072, China

**Keywords:** COX-2, Osteosarcoma, Invasion, Antisense oligonucleotide

## Abstract

**Background:**

Recently, cyclooxygenase-2 (COX-2) has become an important new target in the field of tumor metastasis. However, the relationship between COX-2 gene expression and the behavior of osteosarcoma metastasis is largely unknown. The study is to investigate how antisense oligonucleotides (ODNs) of COX-2 inhibit the invasion of human osteosarcoma cell line OS-732 and their mechanism of regulation.

**Methods:**

A COX-2 antisense oligonucleotide was designed, synthesized, and transfected into OS-732 human osteosarcoma cells. RT-PCR and western blotting were performed to determine the transfection efficiency. A modified Boyden-transwell assay was used to measure the inhibition rate of tumor cell invasion. In OS-732 cells transfected with COX-2 antisense ODNs, RT-PCR was used to examine the mRNA expression of urokinase-type plasminogen activator (uPA) and that of its receptor, uPAR.

**Results:**

Both the mRNA and protein expression levels of COX-2 were significantly reduced when cells were transfected with COX-2 antisense ODNs, which significantly reduced the invasive ability of OS-732 cells in a dose-dependent manner. The expression levels of uPA and uPAR were also significantly reduced (p < 0.01).

**Conclusion:**

COX-2 antisense ODNs significantly inhibited the invasion of OS-732 cells, primarily by decreasing the mRNA expression of uPA and uPAR.

## Introduction

Osteosarcoma is the most common form of malignant bone tumor, and it occurs most often in teenagers. It causes significant harm with poor prognosis, and it is very difficult to cure.

During the past two decades, the emergence of neoadjuvant chemotherapy of osteosarcoma has greatly improved the survival rate of osteosarcoma patients, but nearly half of these patients are not sensitive to chemotherapeutic drugs and die due to lung metastasis within 2 to 3 years.

The majority of patients (80%) have small tumor foci transferred into their systemic blood before treatment; the importance of improving long-term survival in patients by controlling lung metastases through effective drug and gene regulation has been increasingly recognized [1,2]. The invasive ability of tumor cells is one of the important factors in their capacity to metastasize to distant locations in the body [3].

Recently, cyclooxygenase-2 (COX-2) has become a important new target in the tumor metastasis field [4]. Previous studies have found that either COX-2 inhibitors or knockout of the COX-2 gene can inhibit tumor development and invasion [5]. However, the relationship between COX-2 gene expression and the behavior of osteosarcoma metastasis is largely unknown. This project is attempted to investigate the role and mechanism of COX-2 antisense oligonucleotides in regulating the invasion of OS-732 human osteosarcoma cell line.

## Materials and methods

### Materials

OS-732 cells were purchased from Beijing Jishuitan Orthopaedic Laboratory. RPMI-1640 medium (Gibco Company), lipofectamine2000 transfection reagent (Invitrogen), Trizol (Sangon, Shanghai), one-step RT-PCR kit (TaKaRa, Dalian), Matrigel in vitro membrane matrix gel (Peking University), and Transwell cell culture membrane (Coring Costar) were used. Specific primers for COX-2, uPA and uPAR were synthesized by Sangon Shanghai, and COX-2 antibody was purchased from Santa Cruz. Our study was approved by an ethics committee of the Shanghai tenth People’s Hospital, China.

### Methods

#### Synthesis of COX-2 antisense ODNs

The sequence of the COX-2 antisense ODN was 5′GCGGCGACGCTACGAGCGGGCGCGGGACGACGACACG 3′. It was synthesized by Sangon Shanghai. The three phosphate bonds of both the 5′and 3′ ends were modified by sulfide. The 5′end was labeled with fluorescein isothiocyanate (FITC).

#### Transfection of COX-2 AS-ODN

Lipofectin was used for the transfection. The final concentrations of COX-2 AS-ODNs at 100 nmol/L (C1 group), 200 nmol/L (C2 group), 400 nmol/L (C3 group), or 800 nmol/L (C4 group) were used for the transfection experiments. The transfection procedure was as follows: ① OS-732 cells were seeded at 1 × 10^5^ cells/well in a 6-well plate and cultured overnight. ② Measures of 2 -25 μL of Lipofectin liposomes were diluted to 100 μL by serum-free antibiotic-free RPMI-1640 medium in sterile Eppendorf tubes. The AS-ODNs were added to another Eppendorf tube, diluted to 100 μL by RPMI-1640 medium with the same conditions, and incubated at room temperature for 10 min. ③ After 10 min, the above two solutions were mixed gently with a pipette until uniform and incubated at room temperature for 15-45 min to form the liposome-oligonucleotide complex. Then 0.8 mL of antibiotic-free serum-free RPMI-1640 medium was added, bringing the total transfection volume to 1 mL. ④ Cells in the 6-well plate were washed three times with serum-free medium at 4, followed by a final wash with antibiotic-free medium. The liposome-oligonucleotide complex was then added dropwise into cells. The plate was gently rotated to ensure uniform distribution and then returned to the incubator in 5% CO2 at 37°C. ⑤ After 6 h, the liposome-oligonucleotide-containing medium was removed and 2 ml RPMI-1640 medium with 10% fresh fetal calf serum was added. The cells were cultured for additional 24-48 h.

#### Detection of the mRNA expression level in COX-2, uPA and uPAR

The total RNA was extracted with Trizol, and one-step RT-PCR was used to detect COX-2, uPA and uPAR mRNA expression. The 50 μL reaction system contains 5 mmol/L MgCl_2_, 1 mmol/L dNTPs, 0.1 U/μL AMV-Taq, 1 μg of upstream primer, 1 μg of downstream primer, 1 μg of total RNA. Reverse transcription was performed at 50°C for 30 min, and denaturation was performed at 94°C for 2 min. This was followed by PCR for 30 cycles with the following program: 94°C for 30 s, 56°C for 30 s and 72°C for 1 min. COX-2 upstream primer: 5′-TCAAGTCCCTGAGCATCTAC-3′, COX-2 downstream primer: 5′-CATTCCTACCACCAGCAAC C-3′; COX-2 product size is 488 bp. uPA upstream primer: 5′-AGA ATT CAC CAC CAT CGA GA-3′, uPA downstream primer: 5′-ATC AGC TTC ACA ACA GTC AT-3′; uPA product size is 474 bp. uPAR upstream primer: 5′-AGG TGA AGA AGG GCG TCCAA-3′, uPAR downstream primer: 5′-TTC AGG TTT AGGTCC AGA GG-3′; uPAR product size is 553 bp. GAPDH upstream primer: 5′-CTC CCC CTA CTA TCT CTT TC-3′, GAPDH downstream primer: 5′-CAT CTC TCC ATC CCA CTT AAC-3′; GAPDH product size is 330 bp.

#### Western blotting to detect COX-2 protein expression

Cells were lysed by the M-PERTM protein lysate at 4°C and centrifuged at 1500 × g for 15 min. The supernatant was transferred to a new EP tube, where the proteins were concentrated, subjected to SDS-PAGE, and transferred to a nitrocellulose membrane, which was blocked overnight. COX-2 antibodies were used to incubate the membrane overnight, and then the appropriate secondary antibody was added and incubated for 1 h at 37°C after the membrane was thoroughly washed with TBS. The signal was visualized using an ECL detection system.

#### Invasion assay: a modified Boyden-transwell assay was used

1) An 8 μm pore size Transwell microporous membrane was covered with 1 mg/mL Matrigel. Then 500 μL of tumor chemokine was added into the lower chamber, and 100 μL of cell suspension (containing 1 × 10^5^ cells) was added into the upper chamber. The non-transfected control group, the empty vector-transfected liposome control group and the experimental group (divided into 5 subgroups in which the concentrations of COX-2 AS-ODNs were 50, 100, 200, 400 and 800 nmol/L, respectively, in transfected cells) were incubated for 4 h. Then the membranes were removed, fixed by methanol for 5 min and Giemsa stained. Cells on the back of the membrane were counted with a microscope at 200 times magnification. The cells in the middle and four corners were counted three times and averaged. The invasion inhibition rate% = (number of invasive cells in the control group - number of invasive cells in the experimental group)/number of invasive cells in the control group × 100.

## Results

**Figure 1:**
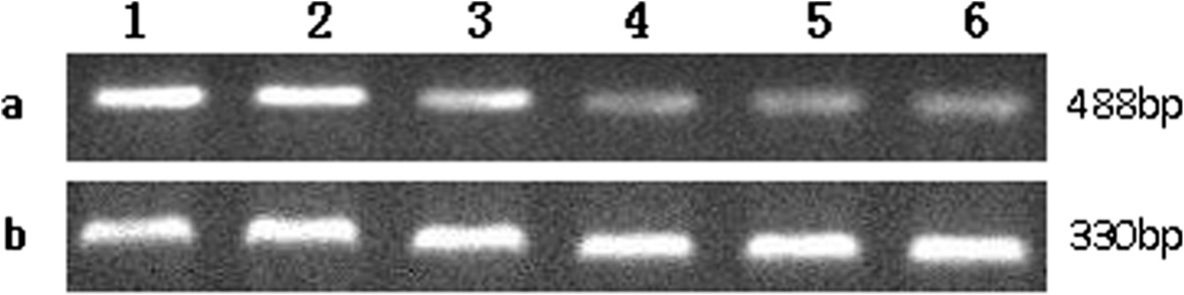
The expression of COX-2 mRNA (12 hours after transfection) a. COX-2 mRNA (488 bp) (1. The control group, 2. Lipofectin group, 3. C1 group: 100 nmol/L, 4. C2 group: 200 nmol/L, 5. C3 group: 400 nmol/L, 6. C4 group: 800 nmol/L); b. GAPDH as an internal control (330 bp).

**Figure 2:**
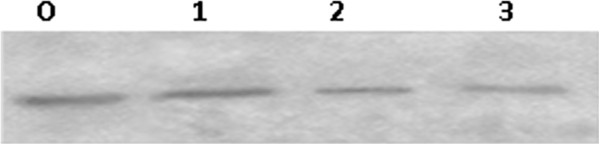
The protein expression of COX-2 by western blotting (0.The control group, 1. Lipofectin group, 2. COX-2 AS-ODN: 100 nmol/L, 3. COX-2 AS-ODN: 400 nmol/L).

The influence of COX-2 antisense ODN transfection on the expression of COX-2 in OS-732 cells. As shown by RT-PCR, the COX-2 mRNA expression levels were gradually reduced in a dose-dependent manner; however, the relationship was not linear. When transfected at a certain concentration (400 nmol/L), the COX-2 mRNA expression declined slowly (Figure 1). In a time-effect relationship, COX-2 antisense ODNs had the strongest effect 12 hours after transfection and then gradually lost potency. Transfection itself did not affect the expression of COX-2, as shown by western blot; however, COX-2 antisense ODNs significantly inhibited the expression of COX-2, and the higher dose of COX-2 antisense ODNs resulted in a greater decrease of COX-2 protein expression (Figure 2).

**Figure 3:**
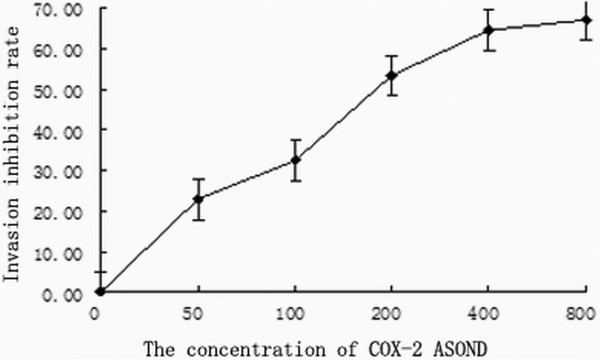
The influence of COX-2 antisense oligonucleotides on osteosarcoma cell invasion.

**Figure 4:**
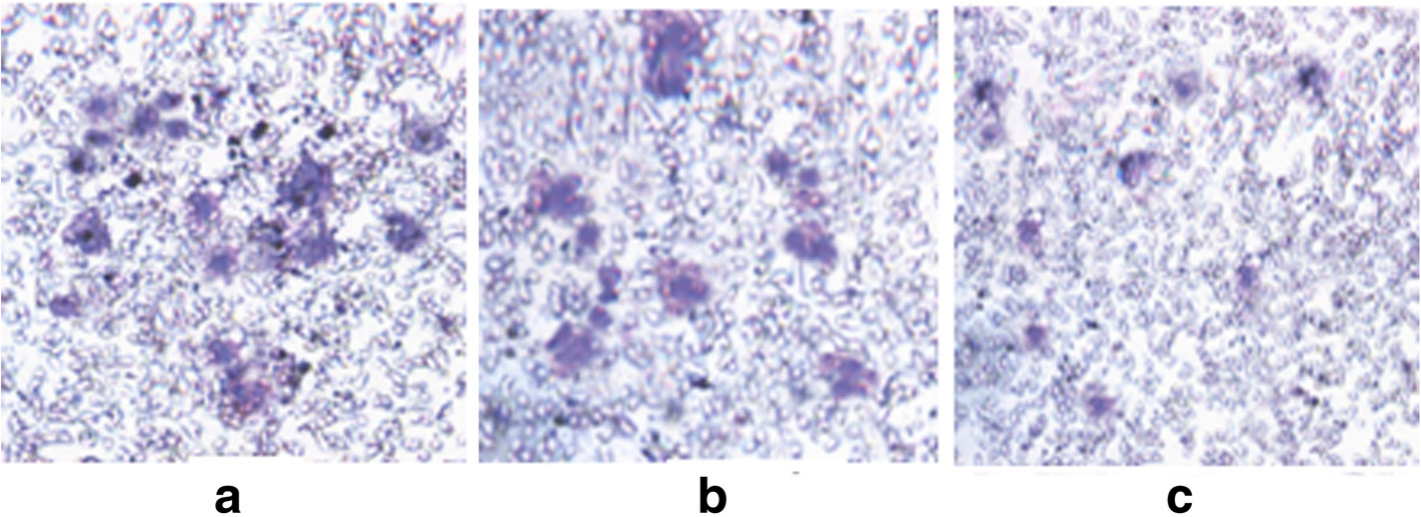
**The number of trans-membrane cells (×200). a**. The control group, **b**. The COX-2 AS-ODN transfection group (100 nmol/L), **c**. The COX-2 AS-ODN transfection group (800 nmol/L).

Transwell invasion experiments showed that the number of trans-membrane cells of the negative control group was 34.42 ± 4.78 at 200 × magnification. That of the empty vector-transfected group was 30.65 ± 5.81, which was not statistically significant different (p > 0.05) from the control group. However, in the COX-2 antisense oligonucleotide transfection group, the invasion capacity of those cells was significantly decreased: the number of transmembrane cells for each subgroup was significantly different when compared with the control group, and the 400 nmol/L and 800 nmol/L groups were the most statistically significant (p < 0.01) (Table 1, Figure 3, 4).

**Table 1:** **The influence of COX-2 antisense oligonucleotides on osteosarcoma cell invasion **$\left(\bar{x}\pm s\right)$

**The concentration of COX-2-ASOND (nmol/L)**	**Penetrating cells (× 200)**	**Invasion inhibition rate %**	**P value**
Blank	34.42 ± 4.78	0	-
Control	31.65 ± 5.81	9.43 ± 2.72	>0.05
50	26.56 ± 3.45	22.85 ± 7.12	<0.05
100	23.26 ± 4.66	32.42 ± 10.26	<0.05
200	17.54 ± 3.36	53.47 ± 9.35	<0.05
400	12.75 ± 3.22	64.67 ± 8.66	<0.01
800	11.86 ± 2.85	67.22 ± 7.52	<0.01

*Note*: Levene statistics was used to confirm the homogeneity of variance between the experimental groups. One-way ANOVA with Bonferroni correction of p-values was used for the movement inhibition ratio.

The influence of COX-2 antisense oligonucleotides on mRNA expression in OS-732 cells.

The specific PCR fragments of uPA, uPAR and GAPDH were amplified by RT-PCR in both the experimental group and the control group.

The optical density ratios of the uPA mRNA amplification products in cells transfected with 0, 200, 400, or 800 nmol/L COX-2 antisense ODNs were 0.89 ± 0.08, 0.75 ± 0.07, 0.62 ± 0.07 and 0.35 ± 0.05, respectively. There were significant differences between subgroups (p < 0.01) (Table 2).

**Table 2:** **Density ratios from the target gene and internal control of samples in each group **$\left(\bar{x}\pm s\right)$

**Group**	**uPA**	**uPAR**
0 nmol/L	0.89 ± 0.08	0.76 ± 0.05
200 nmol/L	0.75 ± 0.07	0.58 ± 0.06
400 nmol/L	0.62 ± 0.07	0.36 ± 0.05
800 nmol/L	0.35 ± 0.05	0.24 ± 0.02
P	<0.01	<0.01

The optical density ratios of the uPAR mRNA amplification product in cells transfected with 0, 200, 400, 800 nmol/L COX-2 antisense ODNs were 0.76 ± 0.05, 0.58 ± 0.06, 0.36 ± 0.05 and 0.24 ± 0.02, respectively. There were significant differences between subgroups (p <0.01) (Table 2).

**Figure 5:**
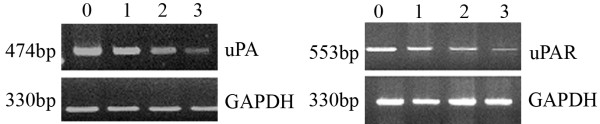
RT-PCR amplification: a. uPA, b. uPAR, group 0, 1, 2 and 3 (COX-2 AS-ODN at 0, 200, 400 or 800 nmol/L).

As can be seen, the expression of COX-2 antisense oligonucleotides decreased the expression of both uPA and uPAR mRNA in a dose-dependent manner (Figure 5).

## Discussion

Cyclooxygenase-2 (COX-2) has been a hot new target of tumor metastasis in recent studies. COX-2 is a rate-limiting enzyme in the synthesis of prostaglandin E2, which is not normally expressed in most tissues. However, COX-2 can be induced by cancer-promoting agents, cytokines, growth factors and hypoxia-inducible factors [6].

In recent years, a remarkable discovery for oncology is that the overexpression of COX-2 is closely related with tumor development. COX-2 is considered to be a new and important target for the treatment of a variety of tumors because COX-2 inhibitors or knockout of the COX-2 gene can inhibit both tumor development and metastasis [7]. Numerous studies [8-11] showed that COX-2 promotes tumor cell invasion and metastasis through various means, including the regulation of downstream genes, in gastrointestinal cancer, lung cancer, breast cancer and other cancers.

Recently, it has been reported that COX-2 is overexpressed in osteosarcoma and is significantly expressed in metastatic lesions [12]. Some selective COX-2 inhibitors inhibited cell growth and induced apoptosis of osteosarcoma cells in vitro. However, the exact role of COX-2 in osteosarcoma invasion is not fully understood. Whether knockout or knockdown of COX-2 expression could inhibit the metastatic potential of osteosarcoma is less well studied.

Malignant tumor invasiveness is an important part of cancer transformation. In the process of tumor invasion, the migration of tumor cells and their distribution to other tissues and organs could be restricted by local organizations, which requires the degradation of the extracellular matrix.

The degradation of the extracellular matrix requires the involvement of a variety of extracellular proteolytic enzymes, with which the urokinase-type plasminogen activator (uPA) and its receptor (uPAR) play important roles [13]. uPA interacts with uPAR at the tumor cell surface, with uPA concentrated on the cell surface. uPA is the initiation factor, activating the conversion of plasminogen into plasmin. Plasmin can directly degrade matrix components or further activate metalloproteinases to degrade the matrix, thereby promoting tumor cell invasion and metastasis [14-16]. The binding of uPA to uPAR also mediates the signal transduction to promote cathepsin B and 92 kDa collagenase expression; to cause non-receptor protein tyrosine kinase phosphorylation of keratin and src family and the activation of the Jak/Stat pathway; and to regulate tumor cell proliferation, differentiation, movement and angiogenesis [17-20].

At the same time, uPAR can be combined with matrix vitronectin and interact with integrins to mediate cell adhesion and movement [21]. Recently [22], uPAR was reported to be a chemokine, which is conducive for tumor cell invasion.It has been proven that the expression of uPA and uPAR was significantly increased in many high-metastatic tumor cells, such as osteosarcoma cells. Higher the expression of uPA and uPAR is associated with worse prognosis and a shorter survival time [23].

In this study, the invasive ability of osteosarcoma cells was detected with the modified Boyden-transwell chamber method. When the concentration of transfected COX-2 antisense oligonucleotides reached 50 nmol/L, the inhibition of tumor invasion was statistically significant; at a concentration of 200 nmol/L, the inhibition rate of tumor cell invasion is 50%, and at a concentration of 800 nmol/L, the inhibition rate reached a plateau.

Molecular biology experiments show that COX-2 antisense oligonucleotides significantly reduced uPA and uPAR mRNA expression in OS-732 cells, and that the effect increased in a dose-dependent manner. Blocking the initial part of invasion could be one of most important mechanisms of COX-2 antisense oligonucleotides in the regulation of the invasive ability of osteosarcoma cells.

The significance of our research is that it has increased our understanding of the relationship between COX-2 gene expression and osteosarcoma cell invasion, which can not only provide research ideas for the inhibition of the metastasis of osteosarcoma cells but also provide a theoretical basis for fighting osteosarcoma metastasis with selective COX-2 inhibitors.

## Competing interests

The authors declare that they have no competing interests.

## Authors’ contributions

XW designed research. MC and XW performed research. LMLwas responsible for statistical analysis. XW drafted the manuscript. FJ revised it critically for important intellectual content. All authors read and approved the final manuscript.

## Authors’ information

Ming Cai is co-first author.
